# An investigation of theory-practice gap in undergraduate paramedic education

**DOI:** 10.1186/1472-6920-9-23

**Published:** 2009-05-18

**Authors:** Rebecca Michau, Samantha Roberts, Brett Williams, Malcolm Boyle

**Affiliations:** 1 Monash University, Department of Community Emergency Health and Paramedic Practice, PO Box 527, Frankston 3199, Victoria, Australia

## Abstract

**Background:**

Bachelor of Emergency Health (Paramedic) (BEH) students at Monash University undertake clinical placements to assist with the transition from student to novice paramedic. Anecdotally, students report a lack of opportunity to practise their clinical skills whilst on placements. The barriers to participation and the theory-practice gap have not been previously documented in Australian paramedic literature. The purpose of this study was to investigate the theory-practice gap for paramedic students by linking education and skill level to case exposure and skills praxis during clinical placements.

**Methods:**

A cross-sectional retrospective study using a convenience sample of second and third year BEH undergraduate students. Ethics approval was granted.

**Results:**

Eighty four second and third year BEH students participated. 59.5% were female (n = 50), 40.5% were male (n = 34). Overall, students most commonly reported exposure to cardiac and respiratory cases and were satisfied with the number of cases encountered during placement. However, over half (n = 46) reported being exposed to < 50% of cases that allowed skills praxis. The most common barrier to participation (34.5%) was the opportunity to participate in patient care and 68% of student's were unsure if paramedics understood their role during clinical placements.

**Conclusion:**

This study demonstrates that the majority of students were satisfied with their clinical placement experience; even though they were exposed to < 50% of cases that allowed skills practice. Identifying these educational barriers will assist in improving the quality and theory-practice gap of paramedic clinical education.

## Background

In Victoria, paramedic education has evolved from post-employment (diploma) to pre-employment (bachelor) education model since 1998 [1]. Paramedic students within the pre-employment model are now required to complete a three year bachelor degree before commencing paramedic employment. This model encompasses an extensive theoretical base, however in contrast to the post-employment model; students are provided with limited 'on the job' skill acquisition via clinical placements. Clinical placements form a fundamental component to undergraduate programs and provide students with a means of developing communication skills, professional socialisation, working in an interdisciplinary team environment, learning professional etiquette and practicing psychomotor skills [2-4].

While clinical placements form a fundamental role in professional development for undergraduate students, a theory-practice gap has been identified across multiple health care disciplines [3,5-8]. This gap between knowledge and practical application can affect professional competence and contribute to difficulties in progressing from student to novice professional [6,9]. The opportunity to integrate theory with practice is currently being affected by a constrained health care system and general shortage of clinical placement opportunities. The scarcity of clinical placements limits training opportunities for students to work with real life patients, which is undisputed as an invaluable learning experience [8]. Placement shortages have derived from limited funding for training, staff shortages, patient availability, competition for placements between health care disciplines and an increasing number of students [10-15]. In addition, this is further compounded with a pressured health care system that can not adequately support student placements and the theory-practice gap has become an evident paradigm [5,16-19]. In particular, international ambulance services have stretched resources [20-23] and in Victoria, ambulance services are unable to meet pre-employment clinical placement demands [8].

Several Australian universities now offer undergraduate paramedic education, including Monash University who provide the BEH. Despite the challenge of scarce clinical placements the course has been designed to maximise clinical placement hours to assist with theory being transferred into practice. First year Monash University BEH students undertake 4 days of observer shifts as an orientation to the paramedic discipline and broader health sector. While second and third year students are expected to actively engage in patient care and complete approximately 140 hours of clinical placement in a rural (Rural Ambulance Victoria) and metropolitan (Metropolitan Ambulance Service) emergency ambulance setting each semester. In addition, third year BEH students also complete clinical placements within a hospital setting in obstetrics, mental health, paediatrics, accident and emergency departments, critical care units and intensive care units to assist with clinical skill development. Furthermore, the BEH program integrates skill praxis via 'real time/life' scenarios and simulations via case-based learning to assist with the theory-practice gap [8]. This approach, whilst offering authentic patient problems, also provides students with learning empowerment and capacity to formulate their own learning outcomes based on previous experiences and critical reflection. Similar pedagogical approaches, such as problem-based learning have been suggested to bridge the gap between theory and practice in other health care disciplines [24].

Little research has evaluated the effectiveness and value of clinical placements for paramedic students, adding to the need for further research in this area, Levett-Jones (2007) correctly points out, that simply sending students on clinical placements does not necessarily guarantee learning or indeed clinical competence [25]. Waxman and Williams (2006) have published student's concerns of "not having enough clinical experience to make a smooth transition from uni student to paramedic student" [p.6, [23]] and that there seems to be a negative mentality towards pre-employment students resulting in students being treated poorly during clinical placements [26]. Boyle et al (2008) also found that although clinical placements were a positive experience within the ambulance setting, students reported unproductive downtime, not being given the opportunity to participate in patient care and that the learning environment was not always supportive [27]. The attitudes of some paramedics towards slightly built females undertaking the physical role of a paramedic was also counter productive for learning [27]. These studies questioned what skills and knowledge paramedic students are being reinforced during clinical placements and whether paramedic clinical placements are as successful as they could be in assisting with the theory-practice gap paradigm. While these studies have importantly added to the body of knowledge in paramedic education, there still appears to be gap in the literature relating to clinical placement education and more specifically theory-practice gap. While commentators continue to describe the difficulties obtaining placements, and potential alternatives in educational delivery [28]. Little discourse has emerged surrounding the notion of how best to utilise the available clinical placements, how to better prepare students for their placements or how to engage students in reflection and reflective practice as described by Boud (1999) and Schon (1987) [29,30]. Reflection is central for students in making sense of clinical dilemmas experienced during clinical placements, particularly when they are engaged in situated or deliberate learning opportunities [29].

The theory-practice gap has not previously been researched in the Australian paramedic discipline. This study aims to investigate paramedic theory practice gap by matching learning objectives appropriate to the students education level to case exposure whilst undertaking clinical placement. Secondly, it aims to examine students' participation levels in patient care according to their clinical scope of practice and to identify factors preventing clinical skills practise during clinical placements. Thirdly, this study aims to document student's perceptions of theory enhancement during clinical placement down time.

## Methods

The 21 item questionnaire was designed for the current study. Questions assessed demographics (4 items), clinical placement cases (4 items), down time during placements (4 questions), expectations of students participation (4 items) and participation in patient care (2 items) (see appendix 1).

The majority of questions required yes/no responses followed by open ended questions to explore qualitative themes. Variations to this included one item for clinical placement cases, one item for down time and two items for participation in patient care. The clinical placement cases item presented students with a list of cases grouped by cardiac (5 cases), respiratory (7 cases), medical (9 cases), trauma (9 cases), mental health (5 cases) and other (11 cases) (see Table 1). Students were asked to indicate which cases they encountered during clinical placements. For down time, a list of activities was provided and participants could respond with either not at all, sometimes or frequently. For participation in patient care, students were asked to rank from 1 (most common) to 5 (least common) for barriers to participation. Similarly, level of participation was assessed by ranking 1 (most common) to 6 (least common).

**Table 1:** the percentage of cardiac, mental health, respiratory, medical, trauma and other cases second (n = 27) and third year (n = 57) BEH students were exposed to during rural and metropolitan ambulance clinical placements.

	**2^nd ^Year Students**		**3^rd ^Year students**	
	
**Type of case**	**Yes**	**No**	**Yes**	**No**
**Cardiac**	**n (%)**	**n (%)**	**n (%)**	**n (%)**

Chest pain	23 (85.2)	4 (14.8)	55 (96.5)	2 (3.5)
Cardiac arrest	6 (22.2)	21 (77.8)	27 (47.4)	30 (52.6)
**Mental Health**				
Attempted suicide/suicide	4 (14.8)	23 (85.2)	29 (50.9)	28 (49.1)
Panic attack	3 (11.1)	24 (88.9)	18 (18.0)	39 (68.4)
Acute psychotic episode	6 (22.2)	21 (77.8)	17 (29.8)	40 (70.2)
**Respiratory**				
Asthma	10 (37.0)	17 (63.0)	42 (73.7)	15 (26.3)
Shortness of breath	14 (51.9)	13 (48.1)	48 (84.2)	9 (15.8)
**Medical**				
Hypoglycaemia	8 (29.6)	19 (70.4)	38 (66.7)	19 (33.3)
Seizure/post ictal	6 (22.2)	21 (77.8)	33 (57.9)	24 (42.1)
**Trauma**				
Motor vehicle accident	8 (29.6)	19 (70.4)	37 (64.9)	20 (35.1)
Fracture	9 (33.3)	18 (66.7)	38 (66.7)	19 (33.3)
Head injury	6 (22.2)	21 (77.8)	20 (35.1)	37 (64.9)
**Other**				
Interhospital transfer	8 (29.6)	19 (70.4)	29 (50.9)	28 (49.1)
Nursing home transport	8 (29.6)	19 (70.4)	32 (56.1)	25 (43.9)
Intoxicated	8 (29.6)	19 (70.4)	31 (54.4)	26 (45.6)
Drug overdose	6 (22.2)	21 (77.8)	29 (50.9)	28 (49.1)
Pain relief	20 (74.1)	7 (25.9)	41 (71.9)	16 (28.1)
Paediatric patient	11 (40.7)	16 (59.3)	39 (68.4)	17 (29.8)

The questionnaire was distributed to second and third year BEH students at the completion of lectures during semester one, 2008. Students were asked to retrospectively reflect on their previous clinical placement experiences when completing the questionnaire. Participation in the study was voluntary and ethics approval was granted from the Monash University Standing Committee on Ethics in Research Involving Humans.

Statistical analysis was undertaken using SPSS (Statistical Package for the Social Sciences Version 15.0, SPSS Inc., Chicago, Illinios, U.S.A.).

## Results

Eighty four participants completed the questionnaire, 32.1% were second year BEH students (n = 27) and 67.9% were third year BEH students (n = 57). The participants were 59.5% female (n = 50) and 40.5% male (n = 34) and 75% of participants were less then 25 years old.

The main cases students were exposed to during clinical placement are presented in Table 1. A wide variety of cardiac, mental health, respiratory, medical, trauma and other cases are being encountered by BEH students whilst undertaking clinical placements. The frequencies of cases seen by students were highly variable between the categories and for the students' year level.

Cardiac and respiratory cases were most commonly reported by students. The most common cardiac case was chest pain (85.2% of second year students and 96.5% of third year students) and cardiac arrest with 22.2% of second year students and 47.4% of third year students having exposure. For respiratory cases, 51.9% of second year and 84.2% of third year students had patients with non-specific shortness of breath (73.8%). Asthma was also seen by 37% of second year and 73.7% of third year students.

For all other cases, students were mainly exposed to cases involving pain and associated pain relief (second year 74.1% and third year 71.9%) and paediatric patients (40.7% of second year and 68.4% of third year students). Approximately 30% of second year students encountered an interhospital transfer, nursing home transport, intoxicated patient or a drug overdose, where as approximately 50% of third year students were also exposed to these case types.

The study also assessed student perceptions of whether learning objectives were being reinforced during placements. The majority of students (79.8%) were satisfied with the number of cases they attended and 94% reported they were exposed to cases that reinforced learning objectives or skills from subjects in the BEH course. Nevertheless over half (n = 46) reported that less then 50% of cases allowed skills appropriate for their level of education to be practiced. Interestingly 59.5% of students performed skills outside of their scope of practice with the most common being drug administration and blood glucose level testing. Intravenous cannulation was another skill performed outside of students' clinical scope of practice; however these students were already practicing this skill in their current part-time occupation e.g. nursing. Over two-thirds of students (67.9%) were unsure if paramedic crews knew what the student's role was during placements despite 72.6% of students reporting they gave the crew information from the university about the skills level and objectives of clinical placements.

Over 70% of third year BEH students reported they were regularly given the opportunity to manage cases during clinical placements. Another 30% regularly assisted the attending paramedic by taking observations (on scene and during transportation) and only a few students (8.8%) reported being an observer (non-participatory) only during some cases. Second year students predominately observed the attending paramedic undertaking patient care (51.8%) and assisted attending paramedic by passing and/or setting up equipment when needed (33.3%). Some second year students also undertook observation of the patients during transport (18.5%).

Additional questions surrounded the notion of why students were not involved or permitted to assist in patient care. Findings suggest the main obstacle to participation was because cases were outside their scope of practice, followed by not being given the opportunity to participate in patient care by the paramedic crew, in other words, to observe in a passive role. Cases involving critically ill patients who were inappropriate for the student to treat and limiting student participation in patient care. Additionally some students also stated they did not participate in patient care due to a lack of confidence.

The amount of 'down time' reported by students on average was 4 hours for Rural Ambulance Victoria (RAV) placements and 1 hour for Metropolitan Ambulance Service (MAS) placements. During down time, students reported frequently reviewing clinical practice guidelines or other university notes and discussed and de-briefed cases with paramedics. Students also reported that they only occasionally practised skills with paramedics; however this was not a frequent event. Other down time activities sometimes undertaken by students were completing clinical placement workbook or reflective journal, reading through patient care records and engaging in recreational activities such as watching television with paramedics. Almost 80% (79.8%) of students also reported that structured activities during 'down time' would be beneficial for reinforcing learning objectives.

## Discussion

This study indicates that for some student's clinical placements is not providing theory consolidation or skills practice appropriate for their scope or level of education. While students were satisfied with the number of cases during placements and believed clinical placements sufficiently reinforced learning objectives, the exercise of matching learning objectives to case exposure and skills practised for education level revealed contradictory findings.

Second year and third year responses were analysed separately to link learning objectives to clinical placement cases. This study was conducted when second year students had completed foundation subjects such as anatomy and physiology and had undertaken reduced clinical placement hours compared with the third years. Their role during these placements was to gain an introduction to life on the road as a paramedic by having an observer role only. This study has shown second year students were participating at an appropriate level by mainly assisting with equipment or observing the paramedic crew undertake case management. In some instances students were participating at a level higher than an observer role. This was also found by Melby (2000) where nursing students undertaking observer shifts with the ambulance were provided with hands on experiences conducting cardiopulmonary resuscitation, oxygen therapy and bandaging [31]. Anecdotally from a university perspective students are encouraged, if appropriate and under supervision, to complete skills not yet learnt at university. This was occurring for over half of BEH students and indicates observing students are being provided with skill practise during clinical placement despite not yet studying these skills at university.

At the time of completing the survey, third year students had covered cardiac, respiratory, trauma and some cases in the 'other' category including pregnancy/birth, pain relief, some aspects of paediatrics patients, unconscious by an unknown cause and drug overdose. Correlating learning objectives to clinical placements cases, the majority had at least one cardiac and one respiratory case during clinical placements. However limited exposure to trauma cases was reported by third year students with in some cases 40% of students not encountering a trauma case. This also reflects the low number of trauma cases seen by paramedics in Victoria [27]. The lack of practising trauma management skills is pertinent throughout Victorian ambulance practice and is not unique to the current study [8]. Additionally, aside from pain relief where two thirds of students had exposure, only half of students had exposure to cases in the 'other' category that had been learnt at university. This study shows that students are not receiving adequate case expose that link to theoretical concepts and additional theoretical reinforcement may be needed during clinical placement to meet learning objectives, particularly for trauma cases. Alternative educational approaches should be considered in bridging this gap; these alternatives might include DVD simulations, virtual simulation wards, teleconference/Internet videoconferencing ward rounds and integration of e-portfolios.

Discrepancies in the case type/number seen by second and third year students can be attributed to the number of hours of placement completed by each student year level. Not surprising, the greater the number of placements the more cases students were exposed to (40 hours for second year students compared to 280 hours for third year students). Consequently, an obvious solution of increasing case exposure would be to increase clinical placement time; however this is not feasible considering the Victorian ambulance services are already unable to support the increasing number of undergraduate students [8]. Further, from 2009 the ambulance services have reduced clinical placement hours from 600 hours to 380 hours per student in the BEH course. (Lord B 2008, personal communication, July 7)

Skill praxis was also suboptimal for third year students. Less than 50% of learnt skills were practised during clinical placements. These findings are similar to an evaluation completed on undergraduate surgical students where 70% of students failed to complete a procedure that linked to their level of education [7]. This raises the concern that students are not receiving adequate skills practice during clinical placement and a review of supplemental skills practice needs to be considered by the university and the ambulance services. This begs the question; does the problem lie with the university or the paramedic industry itself? Is the university curriculum meeting the contemporary needs of the paramedic environment, or is the industry complicated by other issues such as industrial reforms and enterprise bargaining disputes? Without national standardisation of the paramedic curriculum, or standardised graduate attributes, it would seem blame rests with both parties at this present time. Perhaps skill practice sessions during clinical placement down time could be a possible solution, or consideration of increasing patient simulation during academic semesters. This is particularly applicable for rural placements as students reported on average 4 hours/shift of down time. In addition, students were highly receptive to additional activities during down time to supplement learning. This reception to attaining greater professional knowledge perhaps offers an opportunity for further examination.

Skill practice during clinical placement is not just dependant on the frequency and diversity of cases. The student's level of involvement also plays an integral part of whether skills are practised. The majority of third year students had hands on experience with managing cases, yet 30% of third year students were not engaging in patient management. This shows that not all students are participating at the expected level and raises questions surrounding the notion of job-readiness, work-readiness and ongoing ambulance employability.

Barriers to participation were also explored in this study and several themes were identified including factors relating to the student, case nature and paramedic supervisor. For student factors their skill level, motivation and confidence were all identified as variables inhibiting participation in patient care. As expected, life threatening cases also prevented skills practice. There is a delicate balance of providing students with skills practice and compromising patient outcome. Furthermore, paramedic students perceived that supervising paramedics were not always aware of the student's role during clinical placements. This may have limited student participation either due to the paramedic not actively inviting student involvement or the student not feeling supervised during the placement. Supervision during clinical placement has a profound effect on clinical learning and can shape clinical competence [32,33]. Simply providing clinical placement does not automatically ensure learning will occur [34] and these barriers to student participation should be addressed to enhance clinical placement learning. The notion of clinical placement learning provides an opportunity for future examination and pedagogical/curriculum integration. While students currently complete reflective journals during placements, theoretical explanation of i) reflection models, and ii) how reflection can improve learning and metacognition during placements has room for improvement for both universities and industry partners. Simply put, students/educators can not afford to miss any learning opportunities given the current placement shortages and changes to population health.

This study had several limitations. Firstly the results were conducted at one university with a limited sample size and therefore are only representative of BEH students indicating caution should be exercised when generalising these results. Secondly, when exploring themes for level of participation, barriers to participation and activities during down time students were provided with a series of answers to select from and even though students could add additional concepts, designing the retrospective questionnaire in this manner may have biased student responses. Finally, while the questionnaire largely investigated clinical skills whilst on clinical placements, we acknowledge that many other skills beyond psychomotor proficiencies are offered to students during placements, such as attitudes, values and professional socialisation.

## Conclusion

The theory-practice gap in Australian paramedic education was investigated and an overview of the types of cases students being exposed to during clinical placements was outlined. A wide variety and lack of case frequency was reported, however students perceived they were reinforcing theoretical learning objectives during clinical placements. Not all students were exposed to cases that linked to their level of education and students were only able to practice 50% of the skills learnt at university during clinical placements.

This study highlights the need for supplemental skill practise during ambulance clinical placements and identifies the need for strategies to enhance learning during clinical placement such as further education of paramedics to understand the role of students during placements. A smooth transition from novice to graduate paramedic depends on skills and theory development during undergraduate programs.

## Competing interests

The authors declare that they have no competing interests.

## Authors' contributions

RB was the principal author, drafted the manuscript and contributed to the study design and concept and data analyses. SR contributed to the study concept and critically reviewed the manuscript. BW contributed to the study design and concept and critically reviewed the manuscript. MB contributed to the study concept and design and did the statistical analyses.

## Pre-publication history

The pre-publication history for this paper can be accessed here:


